# Biomechanical optimization of different fixation modes for a proximal femoral L-osteotomy

**DOI:** 10.1186/1471-2474-10-112

**Published:** 2009-09-10

**Authors:** Ching-Lung Tai, Weng-Pin Chen, Hsih-Hao Chen, Chien-Yu Lin, Mel S Lee

**Affiliations:** 1 Graduate Institute of Medical Mechatronics, Chang Gung University, Taoyuan, Taiwan, Republic of China; 2 Department of Mechanical Engineering, National Taipei University of Technology, Taipei, Taiwan, Republic of China; 3 Department of Orthopaedics, Tzu-Chi General Hospital, Taichung, Taiwan, Republic of China; 4 Department of Orthopedic Surgery, Chang Gung Memorial Hospital, Chang Gung University, Taoyuan, Taiwan, Republic of China

## Abstract

**Background:**

Numerous proposed surgical techniques have had minimal success in managing greater trochanter overgrowth secondary to retarded growth of the femoral capital epiphysis. For reconstruction of residual hip deformities, a novel type of proximal femur L-osteotomy was performed with satisfactory results. Although the clinical outcome was good, the biomechanical characteristics of the femur after such an osteotomy have not been clearly elucidated. Therefore, this study presents a three dimensional finite element analysis designed to understand the mechanical characteristics of the femur after the L-osteotomy.

**Methods:**

A patient with left hip dysplasia was recruited as the study model for L-osteotomy. The normal right hip was used as a reference for performing the corrective surgery. Four FEA models were constructed using different numbers of fixation screws but the same osteotomy lengths together with four FEA models with the same number of fixation screws but different osteotomy lengths. The von Mises stress distributions and femoral head displacements were analyzed and compared.

**Results:**

The results revealed the following: 1). The fixation devices (plate and screws) sustained most of the external loading, and the peak value of von Mises stress on the fixation screws decreased with an increasing number of screws. 2). Additional screws are more beneficial on the proximal segment than on the distal segment for improving the stability of the postoperative femur. 3). The extent of osteotomy should be limited because local stress might be concentrated in the femoral neck region with increasing length of the L-osteotomy.

**Conclusion:**

Additional screw placement on the proximal segment improves stability in the postoperative femur. The cobra-type plate with additional screw holes in the proximal area might improve the effectiveness of L-osteotomies.

## Background

The hip joint is important for maintaining posture and aiding locomotion. The joint is formed by the articulation between the femoral head and the acetabulum and any alteration in the anatomy of the bony components induces abnormal mechanical forces on the joint [1]. Patients with congenital hip dislocation, Perthes disease or septic arthritis often exhibit a deformed femoral neck and limb length discrepancy. These residual deformities compromise joint biomechanics and cause abnormal loading of the hip joint [2], and result in clinical symptoms: hip instability, limping gait, shortening of the extremity involved, limitation of range of motion in the hip and weakening of the hip abductors [3]. A disease process affecting the physis of the femoral head with continued growth in the greater trochanter will result in a shortened femoral neck, coxa vara deformity and increased anteversion [4]. The upward displacement of the greater trochanter causes poor abductor function and progressively worsens the Trendelenberg gait [5,6].

Various surgical techniques have been proposed but have had limited success in managing femoral neck shortening and the greater trochanter overgrowth secondary to retarded growth of the femoral capital epiphysis [2,5-8]. Although various procedures exist to treat patients with congenital hip dislocation or Perthes disease, residual deformities including leg length discrepancy, hip joint incongruity, proximal displacement of the greater trochanter, and poor joint biomechanics often persist that remain difficult to solve [9]. For reconstruction of these residual hip deformities, Papavasiliou *et al*. [9] performed a new proximal femoral L-osteotomy in sixteen patients with residual hip deformity (coxa vara, coxa breva and high riding greater trochanter). Good results were reported in all patients after a mean of 4.3 years. The surgical procedure not only provided limb equalization but also repositioned the greater trochanter to its normal level. Additionally, the procedure ensured elongation of the femoral neck with subsequent restoration of gluteus medius length. Moreover, engagement of the femoral cortices anteriorly or posteriorly during the distraction restored the proper degree of anteversion of the femoral neck. Another advantage of the L-osteotomy is the repositioning of the femoral head deeper in the acetabulum, which improves the contact between articular surfaces. However, hip spica casts were used in the Papavasiliou study to ensure postoperative stability.

As computer technology advances, the prospects for more realistic modeling of bone diseases are encouraging. Patient-specific simulations of surgical procedures are now feasible, particularly using computed tomography and magnetic resonance imaging (MRI) techniques. Given the geometric nature of residual femur deformity, the FEA model derived from the reconstruction of 3-D CT images may be helpful for objectively analyzing the stresses in structures with complex shapes, loading and material behavior. Finite element analysis models have been applied extensively in orthopedics and have proven effective for predicting musculoskeletal mechanics in unusual circumstances [10,11]. There is also ample precedent for the use of FEA models to elucidate mechanical behavior for a proximal femur osteotomy [12-15]. In 1987, Fyhrie and Carter [12] explored the role of compressive volumetric stress and strain in the early pathogenesis of femoral head necrosis. In 2002, Yang et al. [13] developed a three-dimensional finite element model using a surface modeling technique to assess the stress distribution at various sizes of segmental osteonecrosis. Recently, Chen et al. [14] designed a three-dimensional finite element analytical model for comparing postoperative stability between large cancellous screw fixation and dynamic hip screw fixation in transtrochanteric rotational osteotomy. The Chen study concluded that dynamic hip screw fixation provides better stability than large cancellous screw fixation. The same study also reported the use of computer simulation to investigate the degree to which transtrochanteric rotational osteotomy moves the region of osteonecrotic femoral head out of the weight bearing area. The results demonstrated that posterior rotational osteotomies were more effective for moving the necrotic region out of the weight bearing area during a gait cycle [15]. Since numerous reports have shown good results in analyzing stress distribution and predicting the postoperative behavior of proximal femur osteotomies, a finite element analysis based on various fixation configurations of the L-osteotomy should be conducted due to the promising results of our previous clinical experience.

**Figure 1:**
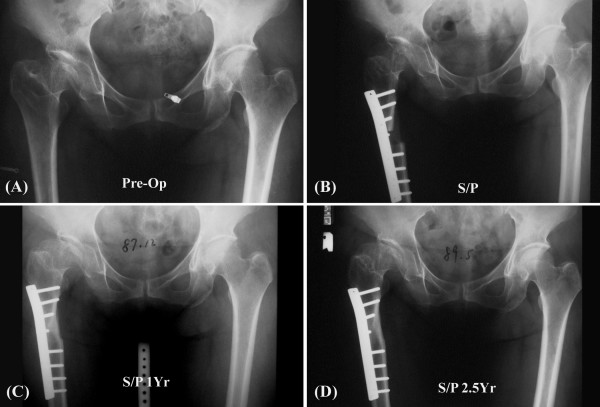
**Radiographs of a dysplasia patient who received an L-osteotomy on Jan. 12, 1998**. (A) Shortening of the femoral neck and relative overgrowth of the greater trochanter, (B) Radiologic appearance after the L-osteotomy and the postoperative results at (C) 1 year and (D) 2.5 years.

In this study, the Papavasiliou method for hip dysplasia was modified by using a longer plate for fixation and achieved good clinical results (Figure 1). Radiologically, elongation of the femoral neck and repositioning of the greater trochanter to its normal level with normal femoral anteversion enhanced hip joint stability. Although this new method of L-osteotomy achieved good results, a thorough understanding and study of its biomechanical properties can further improve the technique and aid preoperative planning. The aim of this study is to understand the mechanical characteristics of the postoperative femur in subject receiving the new type of L-osteotomy with various fixation configurations and different osteotomy lengths. These findings will provide preoperative planning to surgeons in performing osteotomies and thus increase postoperative longevity.

## Methods

### Generation of 3D solid model

This study was approved from the committee of National Science Council of the Republic of China (Contract Number: NSC92-2218-E-182A-001). A left hip dysplasia patient (male, 25 years, 71 Kg, 167 cm) was recruited as the study model for the L-osteotomy. The normal right hip was used as a reference to plan the surgery. 3-D Solid models of both the left proximal femur with hip dysplasia and the normal right femur were created using computed tomography (CT) scan images and Solidworks CAD software (SolidWorks 2004, SolidWorks Corp. Boston, MA, USA). The CT scan images of the intact femur were obtained at 1.25 mm intervals in the transverse planes starting from the femoral head using a GE Hi-speed scanner (General Electric, Milwaukee, WI, USA). The resolution for each of the CT scan image was 512 by 512 pixels, the field of view was 330 mm, and the pixel size was 0.625 mm/pixel. The cross-sectional image files of the femur were transferred to a custom-written automatic contouring program (Caotool) for the detection of the contours between the cortical bone and cancellous bone based on a dynamic density-thresholding algorithm. The parallel-stacked contours were then input into the Solidworks CAD software for the reconstruction of 3-D intact femur solid models.

**Figure 2:**
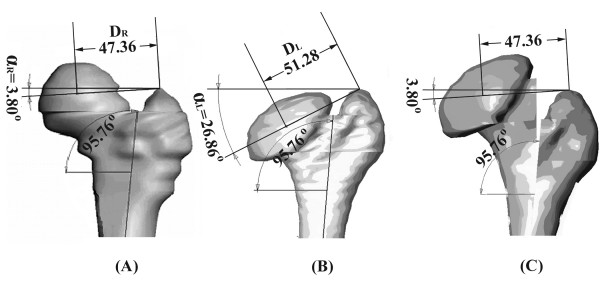
**In L-osteotomy of the deformed left femur, the length and the angle of the intersected line between the femoral head center and the most superior point of the greater trochanter in the normal right femur were considered to be the desired postoperative configuration**. (A) Normal right femur, (B) Left femur with residual deformity and (C) The final resultant configuration after L-osteotomy.

**Figure 3:**
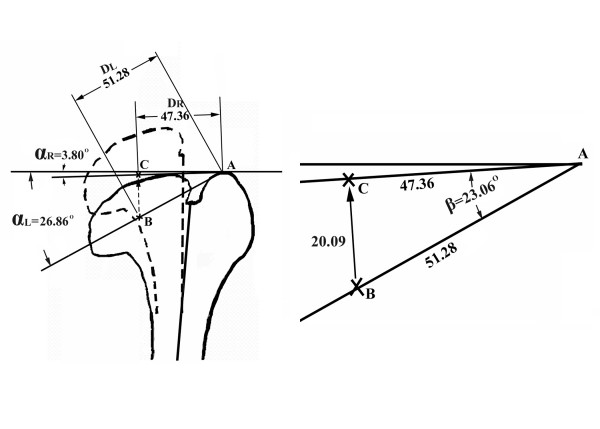
**The lengths of the intersected line between the femoral head center and the most superior point of the greater trochanter in the normal right femur (D_R_) and in the deformed left femur (D_L_) were 47.36 mm and 51.28 mm, whereas the angles of the intersected line with respect to the horizontal line were 3.80° (α_R_) and 26.86° (α_L_), respectively**. The translation of the femoral head center after corrective surgery was then calculated to be 20.09 mm.

For the L-osteotomy of the deformed left femur, the length and the angle of the intersected line between the femoral head center and the most superior point of the greater trochanter in the normal right femur were considered to be the desired postoperative configuration (Figure 2). Based on the generated solid models, a difference in femoral head anteversion between the deformed left femur and the normal right femur was observed, but was considered negligible because it was within 1°. To simplify the simulation procedure, the frontal plane was defined as lying on the plane formed by the femoral neck and shaft axis. The location of the femoral head center was defined as the midpoint of the intersected line between the two points with the largest distance of the contour of the femoral head. Within Solidwork software, a circle with a diameter approximate to the largest distance of the contour of the femoral head was created, the created circle was then moved to fit the two points with the largest distance of the contour of the femoral head. The location of the center of the created circle was defined as the femoral head center to proceed with the subsequent analysis. The line intersecting the femoral head center and the most superior point of the greater trochanter in the normal right femur (D_R_) and the deformed left femur (D_L_) were found to be 47.36 mm and 51.28 mm, respectively, whereas the angles of the intersected line with respect to the horizontal line were 3.80° (α_R_) and 26.86° (α_L_), respectively. The translation of the femoral head center after corrective surgery was then 20.09 mm, as calculated (Figure 3). Based on the above parameters, a biomechanical analysis was performed to evaluate joint characteristics following the L-osteotomy. Finite element analysis (FEA) was used to compare different configurations of screw placement and to determine the mechanical characteristics of the joint with an increasing osteotomy length.

### Generation of finite element model

Based on the solid model of the deformed left femur, four FEA models with different screw placement configurations (P2/D2, P2/D3, P3/D2 and P3/D3; P: fixation on the proximal segment; D: fixation on the distal segment) were created with the osteotomy length kept constant at 126 mm. This length was defined by the roentgenographic measurement of the actual surgery. To investigate the influence of the osteotomy length on postoperative mechanical characteristics, an additional four FEA models with the same number of fixation screws (P3/D2) but different osteotomy lengths (116 mm, 126 mm, 136 mm and 146 mm) were also created (MENTAT 2003, MSC Software Corp., Los Angeles, USA).

**Figure 4:**
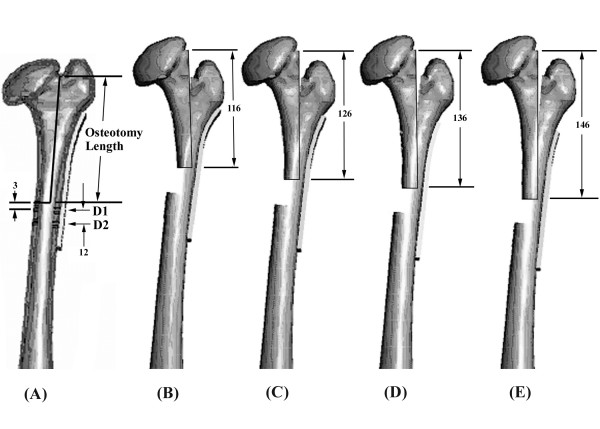
**Femora showing the fixation configuration of the L-osteotomy**. (A) A sagittal osteotomy was assumed from the trochanteric fossa down the middle of longitudinal femoral axis until the defined length of the osteotomy. A horizontal osteotomy was done to unite the medial cortical surface with the distal end of the longitudinal osteotomy. The plate was then fitted to the lateral contour of the proximal femur. The most proximal screw on the lower fragment was positioned 3 mm below the horizontal osteotomy and defined as D1 as described by Papavasilio. The additional fixation screws were placed with a constant interval of 12 mm and defined as D2. All fixation screws were inserted perpendicularly to the femoral contour. Postoperative configurations with (B) 116 mm, (C) 126 mm, (D) 136 mm and (E) 146 mm osteotomy lengths.

A ten-hole plate fitting the bone contour of the lateral surface of the proximal femur was used for fragment fixation following L-osteotomy. The plate was in a width of 65 mm and a length of 150 mm obtained from the measurement of the actual plate (Synthes, Bettlach, Switzerland). The geometry of the screw was assumed to be cylindrical with a constant diameter of 6.2 mm and variable length determined by the location of screw placement sufficient for femoral bi-cortex fixation. The threads and tips of the fixation screws were not modelled in the FEA model in order to simplify the model set-up. A longitudinal osteotomy was made from the trochanteric fossa down to the defined levels of the horizontal cut (116 mm, 126 mm, 136 mm or 146 mm) along the sagittal femoral axis (Figure 4). The distraction of the femur was then performed based on the configuration of the normal right femur, and the length of distraction was found to be 20.09 mm (Figure 3). Following distraction of the femur, the distal end of the upper fragment was then positioned to contact the inner surface of the femoral cortex of the lower fragment, and the fixation plate was placed on the lateral cortex with different screw fixation combinations. The three most proximal holes of the plate were used for screw insertion for upper fragment fixation, and the three most distal for lower fragment fixation, which left the middle four holes of the plate vacant without screw insertion. For all fixation configurations after the L-osteotomy, the most proximal screw on the lower fragment was positioned 3 mm below the horizontal osteotomy and designated as D1 as described by Papavasilio whereas the most distal screw on the upper fragment was designated as P1. For upper or lower fragment fixation, the additional screws were placed on the adjacent hole proximally or distally to the inserted screws and designated as P2 or D2, respectively. All fixation screws were inserted perpendicularly to the femoral contour.

### Loading and boundary conditions

**Figure 5:**
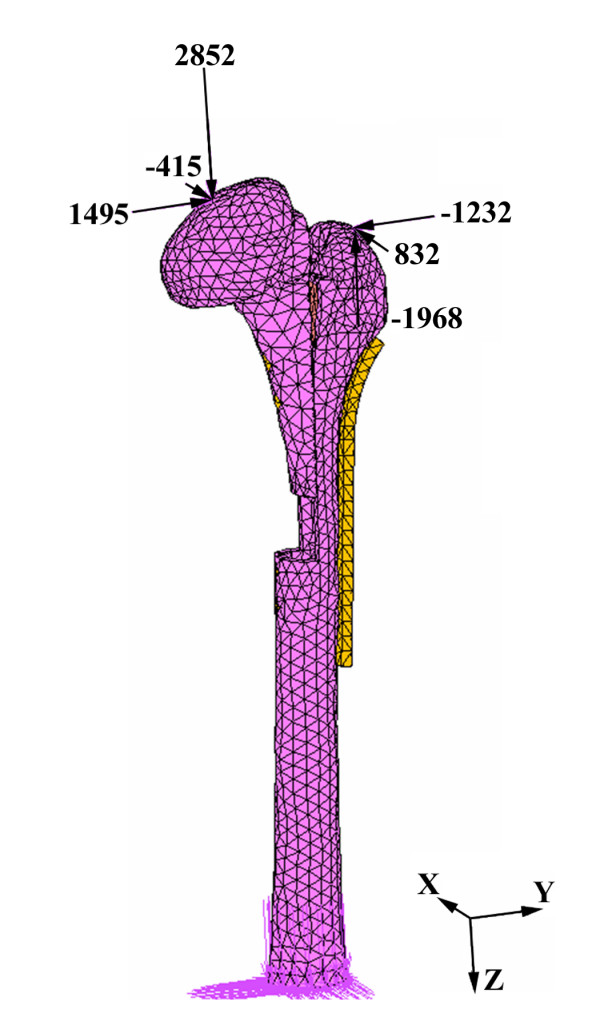
**The single-legged stance loading condition used for the finite element analysis**. The numbers represent the x, y and z components of the applied loadings.

The element type used for all materials in the FEA model was 10-node, isoparametric tetrahedral element. A loading condition simulating a single-legged stance with a 4.54BW (3,246 N) joint reaction force together with a 3.45BW (2,467 N) abductor muscle force was applied on the femur [16]. The loading configuration was shown in Figure 5. Glue contact elements with 1,700 N and 100 MPa were set for the screws/femur interfaces and the longitudinal osteotomized surface, respectively [17]. The distal ends of all models were constrained in all directions as a boundary condition. All material properties were modelled as a homogeneous linear elastic continuum exhibiting isotropic properties. The Poisson ratios and moduli of elasticity used for the cortical bone, cancellous bone and fixation devices were set as 0.3, 0.22, 0.3 and 15.1 GPa, 445 MPa, and 190 GPa, respectively [18,19]. The von Mises stress distributions for each model were analyzed and compared using a commercial finite element package (MARC 2003, MSC Software Corp., Los Angeles, USA).

### Convergence test and model validation

For model verification, a convergence test was used to guarantee our numerical model reached the converged results and no further mesh refinement was necessary. Based on the same solid model, four different FE models with average element lengths of 4 mm, 5 mm, 6 mm and 7 mm were created from the pre-operative femur. The convergences of the FEM models in this study were justified by the total strain energy of the structure. The total strain energy was reviewed for convergence within the four models. The tolerance level was set as the change of less than 5%.

In order to validate the FE model, the experimental results from Shih et al. [20] was used to compare with our FE analysis. In their experiment, the surface strains at the medial and lateral proximal femur under 2000 N vertical loading was recorded in fresh-cadaver compressive testing. To simulate this setting, an additional FE model was created from the intact normal right femur, and the surface strains at the medial and lateral proximal femur subjected to the same 2000 N vertical loading were also analyzed. The experimental and analytical results were compared for the validation of the FEA model.

## Results

### Convergence test and model validation

Results of convergence test demonstrated a less than 5% changes in the total strain energy among four models. The element numbers for four different models with average element lengths of 4, 5, 6, and 7 mm were 92749, 54634, 35031, and 28087, respectively. The total strain energies for each model were 4.131, 4.055, 4.006 and 3.952 J, respectively. The percent differences of the total strain energy compared with that of the finest mesh (element number: 92749) for each of the three models were 1.836%, 3.021%, 4.324% respectively. Although the results indicated that convergence was achieved, the geometry in the medial aspect of femoral neck for FE model with 5 mm element length were somewhat distorted. Therefore, the model with an average element size of 4 mm was chosen as the base model for the creation of post-operative models.

For model validation, the previous study [20] had indicated that the experimental strains at the medial and lateral proximal femur were -1.098 (SD 0.134) and 0.723 (SD 0.139) microstrain, respectively, under a 2,000 N axial loading. In current study, the predicted strains at the medial and lateral proximal femur were -1.211 and 0.742 under a 2,000 N axial loading. The two results were comparable which indicated that our finite element model was reliable for further simulation and analysis.

### L-osteotomy with different numbers of fixation screws but the same osteotomy length (126 mm)

**Figure 6:**
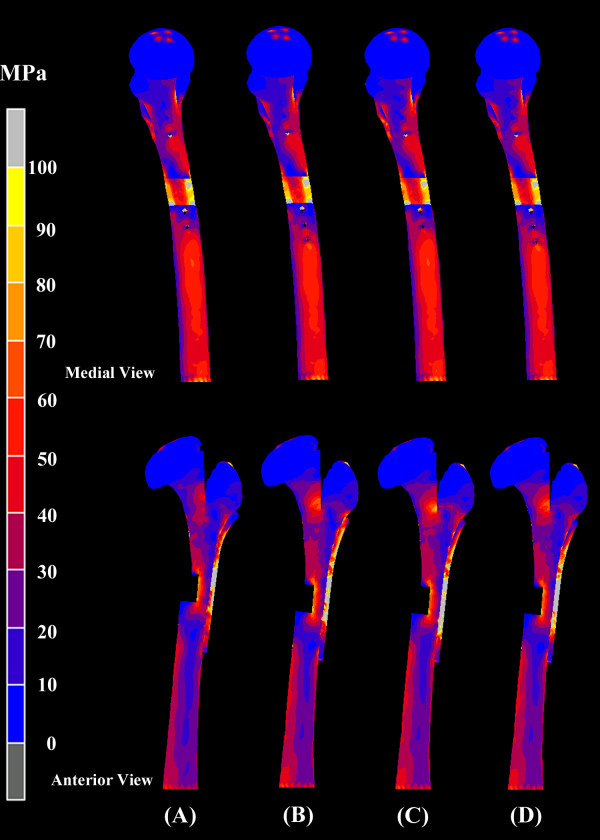
**Medial (top) and anterior (bottom) view of von Mises stress for the reconstructed femora with four different configurations of screw placement but at constant 126 mm longitudinal length of osteotomy**. (A) P2/D2, (B) P2/D3, (C) P3/D2 and (D) P3/D3. (P: Proximal fixation; D: Distal fixation). No obvious difference in von Mises stress was found on the reconstructed femora with different screw fixation combinations.

**Figure 7:**
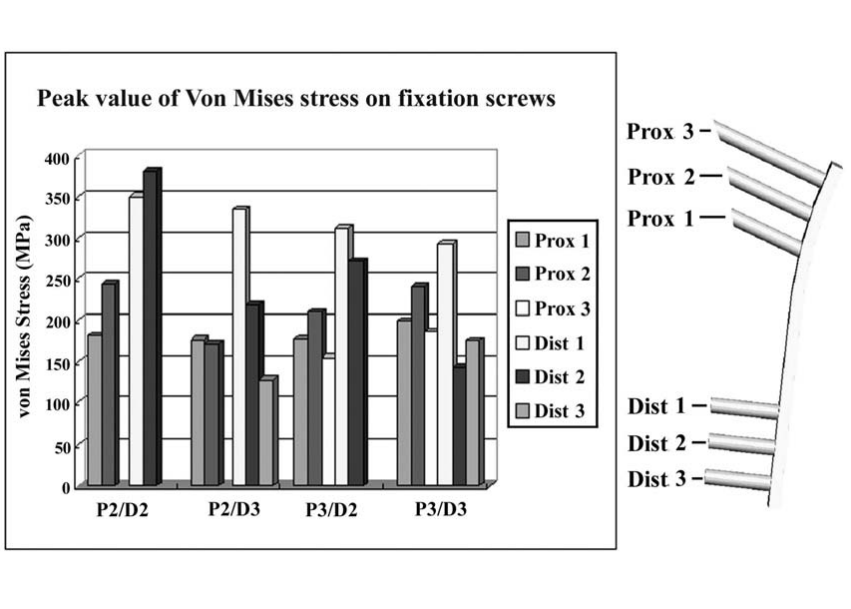
**Peak value of Von Mises stress on screws for femora instrumented with four different configurations of screw placement at 126 mm longitudinal length of osteotomy**. The peak value of von Mises stress of the fixation screws increased with decreasing numbers of screws, and the highest value of von Mises stress was found on the most distal screw (D2) when the femur was instrumented with four screws (P2/D2).

**Figure 8:**
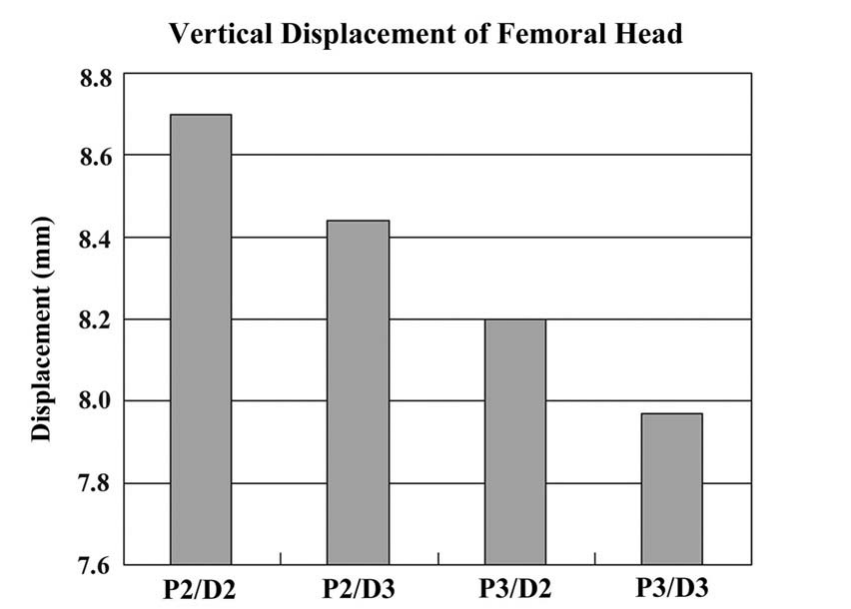
**Vertical displacement of femoral head for femora instrumented with four different configurations of screw placement**. The result indicated that additional screws decreased femoral head displacement (increased stability). A comparison of conditions using different screw placement combinations (P2/D3, P3/D2) revealed that additional screws on the proximal segment achieved more stability of the reconstructed femora than additional screws on the distal segment.

Figure 6 shows the von Mises stress for femora instrumented with four different screw configurations and an L-osteotomy length of 126 mm. No obvious difference in von Mises stress was found on the femora instrumented with different numbers of fixation screws. However, as Figure 7 shows, the peak value of von Mises stress for the fixation screws increased with fewer screws, and the highest value of von Mises stress was found on the most distal screw (D2) when four screws were used (P2/D2). Figure 8 shows the vertical displacement of the femoral head for four different screw placement configurations. The results indicated that with more screws used for fixation, there was less femoral head displacement (increased stability). Comparing the conditions using different screw placement combinations (P2/D3, P3/D2), additional screws on the proximal segment achieved more stability of the reconstructed femora than additional screws on the distal segment.

### L-osteotomy with the same number of fixation screws (P3/D2) but a different osteotomy length

**Figure 9:**
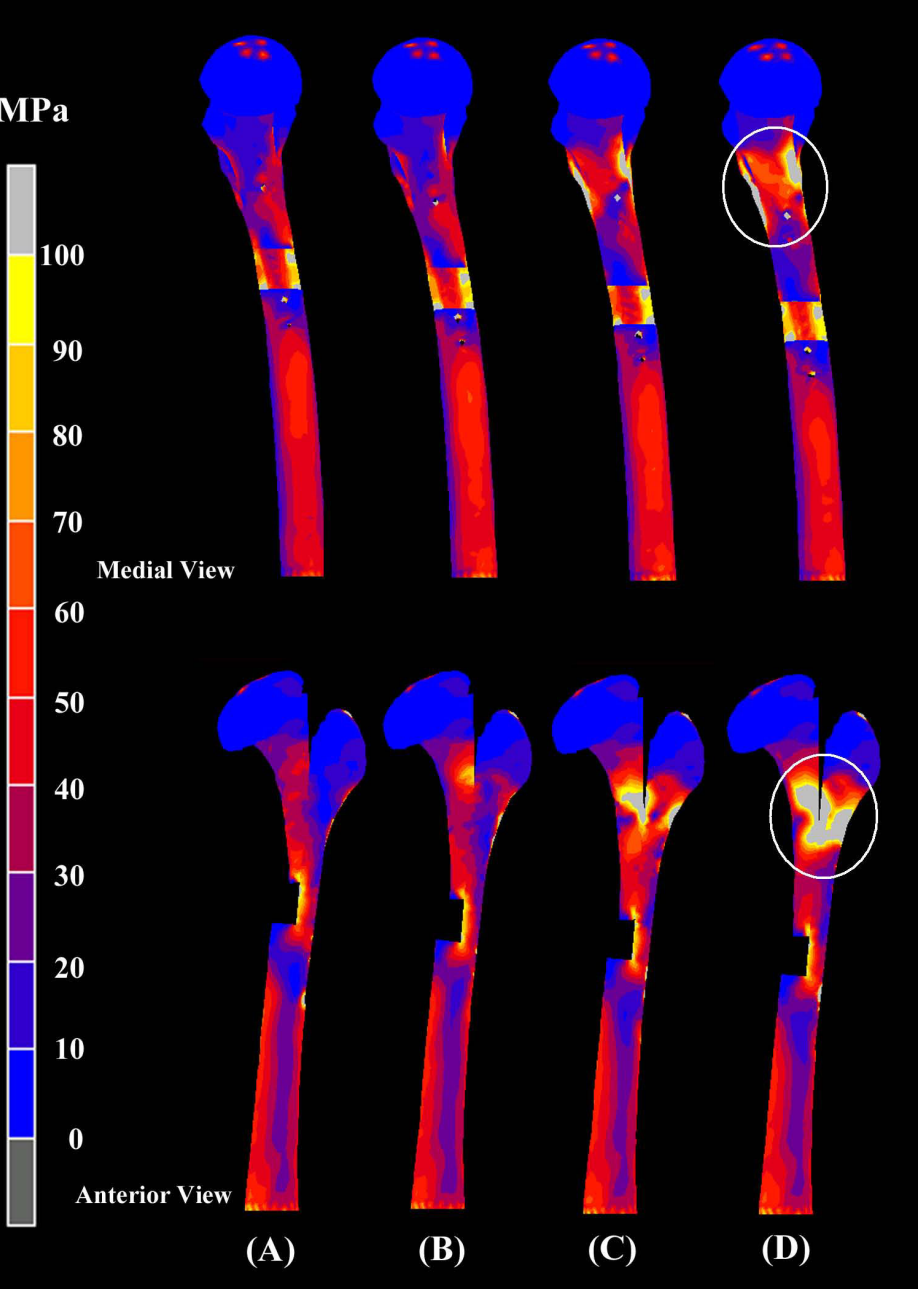
**Medial (top) and anterior (bottom) view of von Mises stress for femora instrumented with four different longitudinal lengths of osteotomy but the same number of screws (P3/D2)**. (A) 116 mm, (B) 126 mm, (C) 136 mm and (D) 146 mm. The von Mises stress around the femoral neck regions progressively increased as the longitudinal length of osteotomy increased from 116 mm to 146 mm.

For an L-osteotomy performed with five-screw fixation (P3/D2), Figure 9 shows the von Mises stress of femora instrumented with four different osteotomy length. The von Mises stress around the femoral neck regions progressively increased as the osteotomy length increased from 116 mm to 146 mm.

## Discussion

Congenital hip dysplasia, Perthes disease and septic arthritis at an early age result in an abnormal gait with limb length shortening, often progressing to advanced osteoarthritis requiring total hip replacement later in life. Many surgical procedures exist for treating these patients, but residual deformities are often present. The L-osteotomy method described by Papavasiliou *et al*. addresses all these deformities by ensuring elongation of the femoral neck, biomechanical improvement of the joint, congruity of the femoral head and equalization of the leg length discrepancy [9]. However, this surgery is a demanding technique, and proper planning for this osteotomy requires a clear understanding of postoperative mechanical performance.

In the present study, the integral femur was sectioned into two separate fragments after the L-osteotomy. The upper fragment contained the entire femoral head and medial proximal femoral shaft, and the lower fragment contained the greater trochanter and the remaining distal femoral shaft. These two separated fragments were fixed into one integral structure by the instrumentation of the fixation devices (plate and screws). It was reasonable to postulate that the fixation devices would sustain most of the external loading applied on the entire femur. As Figure 7 shows, when the L-osteotomy was performed with the same osteotomy length (126 mm), the von Mises stress of the fixation screws decreased with an increasing number of screws because the external load was shared by the additional fixation screws.

Additionally, the femoral head displacement decreased as the number of fixation screws increased (Figure 8). The experimental results further indicated that proximal placement of the fixation screws would be more effective than distal placement for improving stability of the postoperative femur in circumstances when the number of fixation screws is limited. As Figure 9 shows, in an L-osteotomy performed with the same number of fixation screws (P3/D2) but different osteotomy length, the moment acting on the entire structure increased with increasing length, and led to a higher stress concentration on the femoral neck region.

Although an improvement of the integral stability of the postoperative femur was expected by performing an L-osteotomy with more fixation screws, proximal placement of the screw (P3/D2) stabilized the femur more effectively than distal placement of the screw (P2/D3). A Cobra-type plate accommodating more proximal screws would be a good alternative for fixation of the L-Osteotomy.

Finite element analysis is a complementary tool for evaluating the feasibility, efficacy and overall biomechanical characteristics of different surgical techniques. Although clinical trials could summarize general results regarding different osteotomy methods, many fundamental issues still remain controversial and poorly understood. Frequently, scientific inquiries are simulated by the introduction of new surgical techniques. However, systematic investigations are needed to test the treatment principles. Finite element analysis is a noninvasive fracture monitoring technique which can monitor the stress and biomechanical responses of different treatments without clinical influence.

The validation of our model was done with use of the normal right femur, but not the left femur with hip dysplasia. This is because it's almost not possible to access the human cadaveric femur with residual deformities to perform an experiment with L-osteotomy. The validation of FE model in current study is thus conducted using FE model created from the intact normal right femur of the same patient, and the results were compared with those from previous experimental research [20]. In the finite element model, the screw threads and tips are not modeled because FE models with screw threads and tips will result in a large increase of element number and computation time. The simplified FE models without taking threads and tips into consideration may have an impact on the analytic results for local area close to the screw/bone interface. However, we believe these may not cause a global effect on the resultant FE analysis.

Several possible factors affecting the FEA results of the current study must be noted. First, only a single patient with residual deformity was recruited in the present study. Therefore, the results from the FEA might not be regarded as general rules that can be applied to preoperative planning of an L-osteotomy. Importantly, although the results indicated that increased numbers of fixation screws together with a shorter osteotomy length are beneficial for the postoperative performance of the hip joint, preoperative planning should still be individualized based on the extent of the residual deformity of the femur. Second, the FE model was validated based on the intact condition without osteotomy, which may have an impact on the analytic results for the post-operative FE models with osteotomy. However, the boundary conditions including material properties, element types and element length are identical for FE models with or without osteotomy, and we believe that our results provide useful information to orthopedic surgeons performing reconstruction of residual hip deformities with proximal femur L-osteotomy. Third, the only loading condition considered was the single legged gait stance. Therefore, further investigations of the effects of other loading conditions might be necessary in the future. Fourth, the bone plate interfaces were assumed to be fully bonded without considering loosening of the fixation device. Therefore, these FEA results might only be interpreted under a well-fixed condition without implant loosening. Still, attentions need to be paid toward the actual application of this technique. Based on the limitations of this numerical investigation, the model simplifications, such as the material properties, screw geometry and load conditions might influence the accuracy of the mechanical responses and stress distributions obtained in this study. The results from this finite element simulation were based on an objective to provide a way to eliminate the problems encountered with L-osteotomy technique as a clinical treatment substitution.

In Papavasiliou's report, the L-osteotomy not only provided limb equalization but also repositioned the greater trochanter to its normal level [8]. However, good clinical outcomes should not only rely on proper patient selection but also on a good preoperative planning and precise execution of the plan during surgery. In current study, the importance of fixation stability of L-osteotomy was clearly demonstrated by FE analysis. In addition, the significance of fixation screws number and osteotomy length contributing to the stress distribution after L-osteotomy is also highlighted. Since L-osteotomy is a technically demanding procedure and associated with high complication risks, it is suggested that the indication for L-osteotomy should be very strict and that surgical principles should be abided carefully to avoid catastrophic complications.

## Conclusion

This study produced the following findings:

1. The fixation devices (plate and screws) sustained most of the external loading, and the peak value of von Mises stress on the fixation screws decreased with an increasing number of screws.

2. Placement of the screw on the proximal segment rather than on the distal segment enhanced the stability of the postoperative femur.

3. The extent of osteotomy should be limited because a high local stress concentration might occur in the femoral neck region as the L-osteotomy length increases.

## Competing interests

The authors declare that they have no competing interests.

## Authors' contributions

CLT participated in the study design, in collecting the data and drafting of the manuscript.

WPC and CYL participated in the study design. SHC participated in revising critically the manuscript. MSL advised and assisted drafting of the manuscript.

All authors read and approved the final manuscript.

## Pre-publication history

The pre-publication history for this paper can be accessed here:


